# A new concept of treating real-life plastic wastes

**DOI:** 10.1093/nsr/nwaf310

**Published:** 2025-08-04

**Authors:** Jinghong Li

**Affiliations:** Department of Chemistry, Center for BioAnalytical Chemistry, Key Laboratory of Bioorganic Phosphorus Chemistry & Chemical Biology, Tsinghua University, China

The light weight, low cost and adaptability of plastics have made them indispensable, driving global production from 320 million tons in 2015 [1] to 410 million tons in 2023 [2]. Unfortunately, this boom generates immense amounts of low-value plastic waste, which is commonly mixed with municipal solid waste and incinerated or landfilled. This inefficient disposal results in substantial resource wastage and plastic pollution. Reintegrating end-of-life plastics into chemical and material production sections offers a promising path toward sustainability; however, it is hindered by the complex nature of real-world waste [3,4].

**Figure 1.**
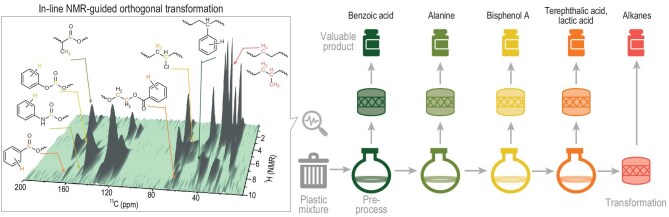
In-line NMR-guided orthogonal transformation of mixed plastics. Reproduced with permission from Zhang *et al*. [5].

In recent research published in *Nature*, Ma and co-workers put forward a novel concept of orthogonal transformation with the aid of the solid-state nuclear magnetic resonance (NMR) technique and catalytic approaches [5]. Based on accurate identification of the characteristic functional groups, the strategy leverages the heterogeneity in the physical and chemical properties of different plastic components, and aims to deal with unknown mixtures comprising a broader range of polymer types for the directional preparation of a variety of valuable products with stepwise extraction–conversion synergy (Fig. 1).

The mixed plastic samples were first analyzed by the solid-state NMR technique (2D ^1^H–^13^C frequency-switched Lee–Goldburg heteronuclear correlation) to make a reliable identification of the functional groups in the intermingled matrix. The orthogonal transformation route was then designed in a targeted manner to get the desired products. For example, starting with 20 g of real-life plastic mixtures composed of polystyrene, polylactic acid, polyurethane, polycarbonate, polyvinyl chloride, polyethylene terephthalate, polyethylene and polypropylene commodities, with integration of solvent extraction, photocatalytic oxidation, catalytic amination, saponification, dehydrogenation coupling and hydrocracking steps, a group of useful chemicals were obtained, including 1.3 g of benzoic acid, 0.5 g of plasticizer, 0.7 g of alanine, 0.7 g of lactic acid, 1.4 g of aromatic amine salts, 2.1 g of bisphenol A, 2.0 g of terephthalic acid and 3.5 g of C3–C6 alkanes.

The NMR-guided orthogonal strategy can effectively analyze and transform unknown waste samples—such as samples consisting of mixed plastic commodities from daily life, post-consumer waste provided by refinery enterprises, discarded plastic parts collected from an automotive repair plant, and samples collected from textile sectors—demonstrating its feasibility and robustness in real-world plastic waste management.

The study proposed a highly adjustable waste resource conversion strategy, under which each specific processing step can be improved and optimized according to the characteristics of the raw materials, the target product requirements and technological progress. Despite its modest overall yield, this proof-of-concept route offers a valuable approach for future applications seeking to optimize the balance between economic benefits, energy and material consumption, CO_2_ emissions and solvent demand, implementation cost and other practical considerations. By leveraging plastic heterogeneity, this orthogonal framework turns diversity from being a problem into an advantage, enabling the construction of product-oriented transformation routes, which provides a paradigm shift in plastic recycling, transforming complexity into modular valorization.
